# The Study of Medicine

**Published:** 1822-12-01

**Authors:** 


*The Study of Medicine.* By JOHN MASON GOOD, M. D. F. R. S. Memb. Am. Phil. Sec. and F. L. S. of Philadelphia. In four volumes, 8vo, pp. 509—935—652—723. London, 1822.

THE construction of a work like this requires an extent of erudition, and an indefatigability of research to which few medical men of the present day can lay claim. When we contemplate the various works which have emanated from the pen of Dr. Good, we must be constrained to acknowledge that his intimate acquaintance with almost all branches of science, literature, and the arts, places him in the very first rank of our learned physicians.* To this we may add, that his zeal and industry are not inferior to his talents and erudition—so that, upon the whole, it is not once in a century that we can hope to find such a union of qualifications for so arduous an undertaking. That a work of this kind was much wanted, is proved by the favourable reception accorded to the compilation of Dr. Thomas—a work of very ordinary pretensions or merit at the best, but now disgraced by English prescriptions for the use of "country clergymen and heads of families"—in imitation of those famous "Reecéan Pandects," which were once the oracles of old women and lay practitioners, after the "primitive physic" of Westley had been gathered to its forefathers. Of a very different cast and complexion is the work before us, the object of which is, to unite the different branches of medical science, physiology, pathology, nosology, and therapeutics, into a general system, so that the whole may be contemplated under a single view, and pursued under a common study.

Without being great sticklers for nosology, we are perfectly ready to admit the immense advantage which the *study* of medicine derives from a nosological arrangement—

* We shall only allude to some of the principal productions of this author—viz. 1. The translation of Lucretius, with copious notes, in two volumes, quarto. 2d. The Book of Job, literally translated from the original Hebrew. 3d. Pantologia, or Universal Dictionary of Arts, Sciences, and Words, by Dr. Good and Dr. Olinthus Gregory. 4th. A Physiological System of Nosology—so well known to the profession. 5th. Self-knowledge. 6th. Sacred Idyls, translated from the original Hebrew. 7th. Sketch of the Revolution in 1688. 8th. Various tracts on Prisons and Poor Houses. 9th. The History of Medicine from the earliest periods. 10th. Essay on Medical Technology; and lastly, the voluminous work before us, containing more than *three thousand pages* of closely printed letter press!

especially when that arrangement is founded, as in the present instance, on a physiological basis, "in which the diseases of the respective functions of the animal frame are connected in classes derived from those functions, and follow each other in the order in which physiologists have usually treated them."*

We agree with our author that, in grouping diseases, not arbitrarily, but in the order of connexion with their appearance in different functions and organs, it is almost impossible to obtain an insight into the nature of any one disease belonging to such groups, without obtaining some insight into the nature of the rest, or tracing out some of the laws of morbid action which are common to the whole. This is a paramount advantage attending a nosological arrangement.† On the particular nosology of our author it is now too late in the day to offer any remarks. It has been five years before the public, and has been adopted as a text-book in various medical schools, as well as by individual writers. Like all new systems of nosology, it requires a new technology—and that is unquestionably an evil. The arrangement of Dr. Good we certainly prefer to every other, though no nosological arrangement has yet appeared, without defects. To the nomenclature too we dare not object, since it is exclusively taken from the Greek, as far as regards his classes, orders, and genera—his authorities, in general, being Celsus and Galen. When he happens to wander farther, he usually supplies himself from Ætius, Cælius Aurelianus, Diascorides, or Aristotle.

A pretty active spirit (as Dr. Good himself observes) of PHYSIOLOGY pervades the whole work—the author prefixing to every class a summary of the most important laws and interesting discoveries of physiology that relate to, or can elucidate the subjects which constitute its scope. He has also occasionally enriched the dissertation by a glance at the more striking analogies of the animal and vegetable world at large, wherever they could add to the illustration.

In the pathological department our author trusts "that nothing is newly started for the mere sake of novelty, or

---

* Preface, p. v.

† That nosological distinctions and minuteness have sometimes been carried to excess, will, we think, be allowed by any one who glances over, for instance, Plenck's Methodical Arrangement of the Diseases of the Eye, in which he will find 119 genera, comprising nearly 600 species, or distinct diseases of a single organ!

Hei mihi! tot mortes homini quot membra; malisque

Tot sumus infecti, mors ut medecina putetur!

controverted from a mere love of disputation." In this department we shall probably have occasion to point out some principles and facts to which Dr. Good's attention does not appear to have been sufficiently drawn.

In therapeutics "the author has allowed himself a liberal range, and has, occasionally, introduced into his materia medica substances that are highly esteemed abroad, though little valued or even known at home, or that seem, without reason, to have fallen into temporary disrepute." Dr. Good thinks, that if the pharmacopœias of former times were occasionally loaded with medicines of trifling importance, the present system of lopping and topping is calculated to make destructive inroads on their boundaries, taking from them much that is good along with some things that might be spared. A work, our author observes, which is erected on scientific principles, should know nothing of the accidental reverses of reputation which befal many of our most powerful remedies—" and still less of the varying, and too often capricious taste of the day." He has, in fact, felt himself called upon by the general voice of the times to range with some latitude over the medicinal stores afforded by art and nature, and to discriminate the respective properties of each, rather than to limit himself to a few leading productions, or to refer to the whole under the general divisions of stimulants, sedatives, cathartics, or other classific appellations.

" Whatever may be the theory or the practice advanced in the ensuing volumes, the author will generally be found to leave nothing upon trust; but to support or illustrate his assertions by authorities which he has endeavoured to give, with some degree of copiousness, from ancient as well as modern times; so as to render the work in a certain sense a summary of the general history of medicine in most ages and countries." Pref. p. xii.

The above passage will shew that any attempt at a regular *analysis* of an elementary work occupying *three thousand pages* of *analytical* matter, would be quite preposterous. Nevertheless, as we consider these volumes eminently calculated to facilitate the study, and improve the practice of the rising generation at least, we shall take a rapid survey of the six great classes into which Dr. Good has divided his subject, by which course we shall have an opportunity of affording our readers a considerable insight into the nature and execution of the work, while we offer such candid commentaries as our experience or studies may suggest.

### Class I. *Cœliaca. Diseases of the Digestive Functions.*

This is the first, and perhaps the most important class of all. It occupies nearly the whole of the first volume of the work. We think Dr. Good was right in beginning with the digestive function, because it is the only one common to all classes of animals from the alderman to the hydatid*—all having an internal cavity of some kind—and all appearing to have a propensity to store it with provision of one description or other.

Preceding the actual diseases of the alimentary canal, we have a neat physiological proem, or sketch, of the organs and functions of digestion—both in man and animals. These proems, which are prefixed to all the classes, are well calculated, not only to enlarge the student's views, but to lead him to cultivate a more intimate acquaintance with comparative anatomy and physiology than he might otherwise be inclined to do. A short extract or two from this portion of the work will shew Dr. Good's manner and style. After alluding to the almost infinite variety of food made use of by different nations, and different classes of society in the same nation, from turtle and venison to fish-oil and potatoes, Dr. Good sums up thus:—

"Man therefore is omnivorous. But he is not the only omnivorous animal in the world; for the great Author of Nature is perpetually showing us that though he operates by general principles, he is in every instance the lord and not the slave of his own laws. And hence among quadrupeds the swine, and among insects the ant, (and more examples might be adduced if necessary,) possess as omnivorous a power as man himself, and feed equally on the fleshy parts of animals, and on grain, and the sweet juices of plants." P. 5.

Dr. Good's extensive reading and retentive memory enable him to enliven the most common elementary details by interweaving curious, uncommon, or illustrative examples, in almost every page of the work. Thus, while shewing that in some animals, as in the zoophytes for instance, the alimentary canal is imperforate, he observes that man himself is sometimes born with this conformation, the office of the natural outlet being vicariously supplied by the urethra, vagina, navel, groin, or other part.

"The most extraordinary instance of accommodation of this kind which I have ever met with in the collections of medical curiosities, is that of a girl, who, from birth, was imperforate both in the anus, and meatus urinarius; in fact in the whole division of the vulva: and who to the age of fourteen, when the account was

---

* Extremes approximate in this as in other instances. Thus the hydatid is a globular membrane, i. e. *all stomach*—and what more or less is the alderman?

written, had regularly discharged her urine by the breasts after the manner of milk, and her feces by a natural vomiting or rejection from the stomach."* 3.

It is known that Dr. Good's first class, cœliaca, contains two orders, enterica and splanchnica, or diseases affecting the alimentary canal, and the collatitious viscera. Odontia, or derangement of the teeth, is the first genus in the first order, and dentition is the first species. Dr. Good introduces a short, but interesting sketch of the natural history of the teeth, before entering on the disorder of teething. We can only make allusion to some points in the treatment of dentition. Dr. Good is an advocate for lancing the gums when there is much irritation, but he thinks the relief is not from the discharge of blood, "but by giving a direct opening to the tooth." In this stage, says he, "if we cannot at once cut directly down upon the tooth, the lancet had better be withheld." In this precept we cannot agree with our author. From very considerable attention to the subject we are quite satisfied that the discharge of even a few drops of blood from the tumid gum gives relief, even if we do not reach the tooth. We therefore have been long in the habit of scarifying the gums repeatedly before the evolution of the tooth, and found that the pain was next to nothing, and the benefit almost certain. We agree with our author that the idea of an indurated cicatrix being left after the use of the gum lancet is quite hypothetical. On the contrary, the recently united edges of the gum, as in all other parts far more readily give way to the process of absorption than if such division had not been effected.

The cutting of the *dentes sapientiæ* often occasions considerable pain, not only in the jaw, but in the corresponding ear, requiring a very free crucial incision through the callous gum, and sometimes a removal of the tooth.

Our author relates some curious instances of those playful attempts on the part of nature to reproduce teeth at a very late period of life, viz. at the age of 60, 80, or 100. Dr. Good attended a lady who cut several straggling teeth at the age of 74, and at the same time recovered such an acuteness of vision as to throw away her spectacles, and read with ease the smallest print.

The section on tooth-ache (odontia dolorosa) occupies nearly twenty pages, and contains a great deal of curious as well as useful information for the student.† In the section on

---

* Samml Med. Wahrnehmung. Band. viii. p. 29.

† We have just heard a gentleman remark that he was rather disappointed in not finding *minute* information on a particular subject, for odontia stuporis (tooth-edge) there is some ingenious physiological reasoning respecting the close reciprocity of feeling at all times maintained between the teeth and the tympanum, from a union of their respective nerves. Hence the teeth often sympathize with the ear, and are set on edge, as it is expressed, by harsh, dissonant, or stridulous sounds. Perhaps the best mode of cure is habitual exposure to the cause of the affection, whereby the too delicate feeling is gradually blunted. "The grating sound produced by filing a saw was probably at one time harsh and abhorrent to the ears of the sawyer; but by being inured to it, he at length hears it with indifference."

No very clear explanation, as Dr. Good observes, has yet been given of the *tartar of the teeth*. Berzelius considers it to be at first hardened mucus, during the destruction of which phosphate of lime is deposited on the enamel of the tooth. The tartar itself, when analyzed, is found to consist of concrete or dried saliva, hardened by its own earthy materials. If suffered to accumulate too much, it loosens and destroys the gums, thus causing a fetid breath. The daily use of a tooth-brush, with any of the ordinary tooth-powders, is, in general, sufficient to keep down this tartar. If it yield not to these, some of the milder acids may be safely employed—avoiding the oxalic, sulphuric, tartaric, and succinic, for *which four acids only*, the lime of the teeth has a stronger affinity than for the phosphoric with which it is combined. From these therefore we ought sedulously to abstain. If the tartar still bid defiance to the other acids, it must be scaled off by instruments.

Passing over a great many genera and species, we shall stop, for an instant, at Genus v. sp. v. FLATULENCY, a troublesome and obstinate complaint. That air is occasionally *secreted* from the mouths of the secernent vessels was the opinion of Hunter, and is far from improbable. In most cases, however, it is merely separated from the materials introduced into the stomach, when they are in the act of fermentation from imbecility of the organ, or its consociate viscera. It is, for the most part, carbonic acid gas that is extricated at those times, and is sometimes prodigious in quantity. Nor need we wonder at this; for, by the experiments of Hales, it appears that a single apple, during fermentation,

---

which he consulted these volumes. The complaint was unreasonable. For *minute* information we must consult *monographs*, or distinct treatises. In a system like this, however extensive, we can expect no more than general information, and references to other and more elaborate works on the particular subjects discussed. *Rev.*

will give out more than six hundred times its bulk of air—and many of the vegetables we use are more flatulent than apples. Bitters are but too often inefficient in giving that tone to the stomach and bowels which prevents the generation of gas, and aromatics give only temporary relief by the discharge of flatus, which is quickly replaced. In several distressing cases of this kind, which have lately come under our notice, we have seen considerable benefit derived from the carbonate of iron combined with rhubarb and ginger. We generally exhibit ten grains of the iron, three of rhubarb, and three of ginger, twice or thrice a day, in any convenient vehicle, keeping the patients on plain food, and enjoining them to use but little drink.

Dr. Good, after laying before us a great deal of information on this subject, collected from various quarters, concludes with the notice of two remedies which, he says, not only afford benefit at the time, but have a tonic virtue which tends to correct the disorder radically. The first of these is the tincture of the rose wood, or rhodium lignum of the old writers, made by macerating four ounces of the wood in a pint of spirit. "It proves a warm, balsamic, and pleasant cordial in doses of from twenty or thirty drops to a drachm." The second remedy is the etherial oil, as it is now called, or oleum vini, as it was formerly designated, and which is found in the residuum of sulphuric ether. It has a strong, penetrant, and aromatic odour, and readily dissolves in alcohol or ether.

"In the current Pharmacopœia of the London College, this anodyne is imitated in the preparation called compound spirit of ether, the only form in which the etherial oil is employed as an ingredient. For the purpose I am now speaking of, however, it should be dissolved, and in double the quantity contained in the preceding preparation, in the aromatic spirit of ether, the sweet elixir of vitriol of the old dispensatories; in which case it combines its powers with the fragrant and valuable spices of this preparation, and becomes a very grateful carminative and exhilarant." 136.

The section on dyspepsia (gen. v. sp. iii.) is copious, interesting, and instructive. This is justly represented as a hydra-headed monster—"a multiform combination of maladies of which dyspepsy is the general expression." It is a vice having its seat not always or solely in the stomach, but occasionally in one or all of the circumjacent viscera—especially the liver. The etiology of this proteiform malady is ably traced out by our author, as well as its secondary effects on the system at large—especially on the lungs, where it imitates phthisis, and has been well delineated by Dr.

Wilson Philip. The causes may, Dr. Good thinks, be contemplated under two heads—local and general:—"under both which they are still further resolvable into the two opposite extremes of deficient and excessive stimulation."

The *local* remote causes are indulgence in sedative or diluting substances, or stimulating and acrid materials—excesses in eating or drinking—or rigid abstemiousness, and protracted periods of fasting. The *general* remote causes may be classed under the heads of indolent and sedentary lives—intense study, "not properly alternated with cheerful conversation"—immoderate libidinous indulgence—too great muscular exertion—and last, not least, late hours, and spirituous liquors. Our author insists much on the advantages of cheerful conversation, without which, even exercise itself will be of little avail.

"For the mind, accustomed to a certain track of intellectual labour, will otherwise relapse, even while riding or walking, into the same habitual course, be dead to the most fascinating prospects around it, and become exhausted by its own abstraction. And it is to characters of this kind, perhaps more than to any other, that the amusements of a watering-place promise ample success; where the general bustle and hilarity, and the voluntary forgetfulness of care, the novelty of new scenes, and new faces, and new family anecdotes, and the perpetual routine of engagements that fill up the time with what would otherwise be trifles and frivolities, reverse the mischievous order and monotony of the past, break the sturdy chain of habit and association, and give leisure to the worn-out sensory to refresh itself." 153.

But where dyspepsy has resulted from the fashionable follies and dissipation of a town life, a change of residence to the coast, will be a change of place, but not of life.

"A total retreat from the world, the unbroken seclusion of a remote hamlet, the sober society of a few intimate friends, simple meals, and early hours; instead of close and heated rooms, crowded and motley routs, costly feasts, and midnight madrigals, are what are specially called for in this instance, but are not always to be met with in the resort of a watering-place." 154.

The fact is, that London is not the eternal city—at least on the Thames, for she is now alternately on the banks of a river, and the borders of the ocean. Yet we doubt much whether the secluded hamlet and sober society depicted by our author would have much attraction for the fashionable hypochondriac, worn out by unnatural hours, pampering diet, stimulating drinks, and sensual excesses of all kinds. It would be like putting the habitual drunkard at once upon a milk and water diet. They would both be eaten up with the horrors of their own *feelings*! Nothing is more fortunate for patients under such circumstances than a smart attack of some acute disease, under which, strange as it may appear, they will better bear the transition to rigid abstemiousness than when in their ordinary conditions of health.

Our author lays down judicious rules for the guidance of the dyspeptic invalid—limiting the stomach to such food as it will most easily digest—quickening the action of the bowels by gentle aperients—and augmenting their tone by warm bitters and tonics. Magnesia appears to be a very favourite remedy of our author for the correction of acidity—while for the nidorous or fetid eructations the mineral or vegetable acids must, of course, be employed. Dr. Good thinks that charcoal has not lately been used for the purposes in question to the extent it deserves. It is a powerful corrector of putridity—and may be taken in the dose of twenty or thirty grains twice or thrice a day. For the intestinal torpor, nothing, Dr. G. thinks, stands higher than rhubarb uniting an aperient and tonic power. It is often necessary, however, to combine it with such a proportion of extract of aloes, jalap, or colocynth, as may be sufficient to keep up a regular action in the bowels. Ipecacuan, in small doses, is sometimes, but not very generally, useful. "The blue pill will generally answer better," and it is chiefly, he observes, to be employed where we have reason to apprehend that the pylorus or liver is affected. For gastrodynia, a very common concomitant on dyspepsy, we have often seen advantage from the oxyde of bismuth, in doses of five grains thrice a day. Myrrh is strongly recommended by Dr. Good as a warm tonic for the stomach. When dyspepsy is engrafted on an hysterical or hypochondriacal diathesis, we may indulge, he thinks, in stimulants of a warmer character, as camphor, assafœtida, the alliaceæ, the spicey aromatics, and even capsicum. The quassia, gentian, and columbo, offer the best bitters, if we except the chamomile, uniting a warm essensial oil with the bitter. If used in infusion, the vessel should be closely covered, and the menstruum not kept longer than an hour on the flowers. But we must refer to the work itself for many pages of judicious advice respecting medicines, diet, and the employment of time.*

---

* Dr. Good does not mention the dandelion in this section. We have found it very useful, combined occasionally with chamomile, cascarilla, or rhubarb. It increases the biliary and urinary secretions, has an aperient effect on the bowels, and improves the digestion. We generally use the infusion—three ounces of the sliced fresh root to a pint of boiling water, with a little ginger or other spice—sometimes with cream of tartar.

In the section on ileus and colica pictonum, our author has, with his usual industry, collected a great deal of scattered information. We observe, however, in this, as well as in many other instances, that Dr. Good has not brought down his pathological researches to the present moment, which he might easily have done, and certainly ought to have done. Thus he has not alluded to the able observations of Abercrombie on Ileus—nor to the valuable paper on Ileus in the French Dict. des Sciences Medicales, vol. xxiii. Of these papers, however, we gave an extended account in the second volume of our quarterly series, for April, 1820, page 613 *et seq*. In the colica pictonum, or rachialgia, as it is termed in Dr. Good's Nosology, he considers the calomel and opium plan of treatment as far superior to any other that has yet been proposed.

We observe that Dr. Good divides cholera into three species—cholera biliosa—flatulenta—and spasmodica. He acknowledges that it is only in the first species that there is any evidence of an *increased* secretion of bile:—in the two last, indeed, there is, he confesses, more proof of suppression than increased secretion. We have seen numerous cases of all these species, and we confess that our observations have led us to believe that even the common cholera or bilious vomiting of this country, in autumnal seasons, is not *caused* by an increase of the biliary secretion, the latter only taking place in the course of the disorder—principally, perhaps, from the action of vomiting itself. Let a person take an emetic in perfect health. He first ejects the contents of his stomach—then glairy or watery fluids—and, after straining a while, bile comes off in greater or less quantity. Whoever has experienced cholera in his own person, or narrowly watched it, *from its very commencement*, in the persons of others, will be forced to confess that the above succession of events is precisely what takes place in cholera. Nor is this assertion contravened by the descriptions of our best writers, ancient or modern. True it is, Celsus tells us that bile bursts forth both upwards and downwards—bilis supra infraque erumpit. Well; but what kind of bile?—primum *aquæ* similis, interdum alba, &c. Will any modern physician contend that the *white and watery matters* first brought off the stomach are *bile?* We fancy not. The fact is, that while Celsus derives cholera from χολη and ῥεω, literall *bile flux*, Trallian considers it as χολας and ῥεω, which is literally *intestinal flux*. Sydenham is generally quoted in support of the bilious theory; but the English Hippocrates does not even mention the word bile in the description of the disease. "Adsunt enim vomitus enormes, ac *pravo-*

*rum humorum* cum maxima difficultate et angustia per alvum dejectio," &c. p. 175. Areteus gives a more minute description of the disease than either Sydenham or Celsus, and he makes no mention of bile at the beginning of the disease. His description is truth itself. "Supra enim per vomitum erumpunt, *quæ in ore ventriculi, et gula congesta fuerant.* Infra dejiciuntur humores in ventriculo intestinisque natantes. *In primis* quæ evomuntur aquæ similia sunt:*—quæ anus effundit, stercorea, liquida, tetrique odoris sentiuntur. Siquidem longa cruditas id malum excitavit, quo si per clysterem eluantur, *primo pituitosa*, mox biliosa feruntur." Now let us see how the above description agrees with the best modern accounts. Parr expressly says, "the matters voided are at first the remains of the food; and *afterwards* bilious fluids." In Rees's Cyclopedia the article cholera, we believe, was written by Dr. Bateman, and is a very excellent composition. It states that "the bowels are seized with griping pains, and the stools, which are at first thin and *watery*, as in common diarrhœa, are passed frequently. The stomach is seized with sickness, discharges its contents, and rejects what is swallowed. In the course of a *few hours* the matter vomited, as well as that which is discharged by stool, appears to be pure bile, and passes off both ways in considerable quantities." Now let any unprejudiced person contemplate these descriptions, and say whether there appears any foundation for the theory which attributes the vomiting and purging, in other words, the disease, to the presence of bile. It is now acknowledged, though the statement was long laughed at, that in the worst forms —that is, in two species out of three, superabundance of bile is quite out of the question—on the contrary, that the biliary secretion seems lessened and often entirely suppressed. Yet such is the tenacity with which we cling to preconceived doctrines, that because a discharge of bile takes place in the course of one species, (the mildest of all,) we still continue to rank the inordinate secretion of that fluid as the proximate cause of the three, in our systematic works! To us it appears that various causes, many of which we are quite ignorant of, produce an orgasm or violent irritation in the

---

* We see that Celsus evidently copies Areteus, but not correctly—for Areteus makes no mention of bile, at the commencement of the symptoms, and there can be little doubt that Areteus was the practical physician, not Celsus, who is merely a compiler, and is not generally considered as having been a professional man at all. In fact, Celsus has reversed the description of Areteus, and placed the eruption of bile as the *first* symptom, whereas the Greek author makes it the *last*.

primæ viæ, the natural consequence of which is a discharge, upwards and downwards, of whatever the primæ viæ contain at the time—and that this orgasm continuing, an inordinate secretion of bile takes place, as well as an inordinate gastric and intestinal secretion, which inordinate secretion has nothing to do with the etiology of the orgasm itself—on the contrary, we believe it to be a salutary or remedial effort of Nature to remove the disease or its cause. We grant that, at the times when cholera is most prevalent, there is generally an irritable state of the biliary as well as of the gastric system, and consequently that they are both disposed to fall into violent and irregular action. We cannot regard the three species of cholera, as divided by Dr. Good, in any other light than as three grades of the same disease, differing only in violence and in danger—or rather, we should be disposed to say, that the three species depend on the degree of force or concentration in the exciting cause, whatever that may be, aerial or terrestrial, or both combined, which we believe is the case.

Of the epidemic cholera of India, Dr. Good has given as full an account as the documents before the public, and the liberality of Sir James M'Grigor in giving Dr. G. access to the army returns, would permit. We do not see that any new light whatever has been thrown on the etiology of the disease during the prevalence of the epidemic, nor has any important novelty or improvement been devised in the treatment.

In the genus HELMINTHIA, or *invermination*, as our author emphatically terms it in his own tongue, Dr. Good has, as usual, accumulated a great deal of curious information scattered through hundreds of tomes and ephemeral journals. He combats the doctrine of equivocal generation, so warmly espoused by his favourite ancient philosopher Lucretius. We now, he observes, know that an incipient stage of putrefaction, or a very short quiescence and exposure of animal fluids to a warm atmosphere, is sufficient to load them with animalculæ of some kind or other, "not, indeed, by fortuitously converting the constituent and decomposing principles of such fluids into the simple forms of microscopic life, but rather by affording to some few of the myriads of invisible ovula with which the atmosphere swarms, and which it may convey to them, the proper nidus, or the quickening stimulus they stand in need of."

"That the atmosphere is freighted with myriads of insect-eggs that elude our senses; and that such eggs, when they meet with a proper bed, are hatched, in a few hours, into a perfect form, is clear to any one who has attended to the rapid and wonderful effects of what, in common language, is called a blight upon plantations and gardens. I have seen, as probably many who may read this work have also, a hop-ground completely overrun and desolated by the aphis *humuli*, or hop-green-louse, within twelve hours after a honey-dew (which is a peculiar haze or mist loaded with a poisonous miasm) has slowly swept through the plantation, and stimulated the leaves of the hop to the morbid secretion of a saccharine and viscid juice, which, while it destroys the young shoots by exhaustion, renders them a favourite resort for this insect, and a cherishing nidus for the myriads of little dots that are its eggs. The latter are hatched within eight-and-forty hours after their deposit, and succeeded by hosts of other eggs of the same kind; or, if the blight take place in an early part of the autumn, by hosts of the young insects produced viviparously; for, in different seasons of the year, the aphis breeds both ways." 293.

The two grand indications of treatment are first to expel or destroy the worms, and secondly, to strengthen the alimentary canal in order to prevent their reproduction. These two indications may sometimes be pursued simultaneously.

"By having the bowels loose, we prevent the accumulation of the slime in which the worms burrow: and if we have reason to believe that such accumulation has taken place, the best plan is to give active purges, as calomel, jalap, scammony, gamboge, or an intermixture of these, for its removal; and having thus, as far as we are able, exposed the naked bodies of the worms to the action of anthelmintics, we should proceed with the latter without loss of time." 313.

The list of anthelmintics is long enough to convince us that no one of them is possessed of any very strong specific powers. Dr. Good has reviewed the principal of them, dividing them into two classes—those which dislodge or destroy by a mechanical action—and those by a narcotic or other internal means. Among the former we may reckon drastic purgatives, including oil of turpentine, tin-filings, crude quicksilver, mild lunar caustic, cowhage, &c. In the latter, we may place the male fern, hellebore, tansy, savine, rue, santonicum, tobacco, &c. We think Dr. Good has dealt rather freely with the rectified oil of turpentine. "A child of ten or eleven years old may take an ounce without any evil effect in ordinary cases." We have lately exhibited this medicine to a series of young folks, from three to fourteen years of age, in pretty active doses; and though the ultimate effects were excellent, we had reason to be several times alarmed at the primary operation of the oil—not so much from the catharsis as from the intoxicating power of the remedy.

Dr. Good thinks that there are some medicines which may be regarded as specific vermifuges—that is, as acting on the worms by some simple quality which proves highly poisonous to them without affecting the bowels. The chief of these he considers to be the bark and shoots of the cabbage-tree (areca oleracea) and the male fern. The former has been used in infusion, syrup, or powder. It only kills the worms, and consequently it is necessary to exhibit purgatives afterwards to bring them away. Of the male fern Cullen entertained no great opinion, though it is still extolled in Germany.

The section on "Proctica Spasmodica," or "Spasmodic Stricture of the Rectum," is defective in recent information—a defect which we fear we shall often have occasion to regret in the course of our survey of these volumes. In the 5th volume of the Medical Transactions of the College, Dr. Baillie has given a description of this disease, which is quoted freely by Dr. Good, who relates an interesting case that fell under his own observation, and which has now continued six years, without any material benefit from medicine. Our readers are aware that we have given in our last number a full account of M. Boyer's able paper on this subject, published more than four years ago in the Journal Complementaire, and noticed in most of the English journals of that time. It is a great pity Dr. Good did not take that paper for his text, as not only affording a more correct view of the disease pathologically, but as pointing out an operation which almost invariably cures the complaint—we mean a division of the sphincter of the gut. We have seen but one exquisitely marked case of the disease, in a medical gentleman, 42 years of age, of great mental and corporeal activity, but long subject to hæmorrhoids. It precisely answered the description by Drs. Baillie, Boyer, and Good; and after harrassing the patient for more than two years, it has nearly given way, at least it now gives no pain, by a constant and unremitting use of tepid water enemata before every motion. This is a far better plan than giving laxatives by the mouth, and completely secures the irritable stricture of the sphincter from the annoyance of the passage of hardened fæces—a source of irritation which inevitably keeps up the original disease. On this subject, however, we have said enough in our last number.

On the "Proctica Callosa," or callous stricture of the rectum, Dr. Good has mustered as much respectable information as could be compressed in the short space of six pages, which is all that is dedicated to the complaint. We do not see any thing either novel or objectionable in this section.

The fith species of genus xii. is "Proctica Marisca," the author's designation of hæmorrhoids. It is divided into four varieties, the cæca, mucosa, cruenta, and caruncularis. Rather better than ten pages are devoted to the subject of piles. We think Dr. Good has not investigated this disease in proportion to its importance—especially in a constitutional point of view, of which indeed he takes very little notice, excepting as it is connected with a gorged state of the liver. We are sorry that Dr. Good did not consult the very excellent monograph of Dr. Montegre of Paris, inserted in the 20th volume of the *Dict. des Sciences Medicales.* This work was reviewed by Sir Charles Morgan, in the 53d number of the Ed. Journal, and fully analyzed by us in the 2d number of our Quarterly Series for October, 1818.

The treatment of hæmorrhoids divides itself naturally into local and general means. The general means are such as correct the morbid state of the constitution, and especially the digestive function, which is very commonly in fault. General alteratives—the blue pill, sulphur and supertartrite of potash, and electuary of senna, are the best medicines, with or without taraxacum or sarsaparilla. But a great deal of attention should be paid to the state of the constitution generally, in order to discover whether there be erratic gout, or other constitutional diathesis; or whether the hæmorrhoidal functionary movement be vicarious of some other disease. These things should be well weighed before we attempt to repel the anal determination by astringents, as nutgalls, &c. or by the local application of cold, whether in the form of glysters or externally. In this complaint, as in spasmodic constriction of the sphincter, no local means can compare with the employment of lavements, so as to secure the parts from irritation in the passage of the fæces. This process alone would go far to cure the most obstinate cases. Where there is nothing in the constitution to contra-indicate the measure, we have always found very quick and effectual relief from the following ointment, spread thick on a piece of lint, and kept on the painful and protruded piles by means of a handkerchief or T bandage.

℞. Cerat. superacet. plumb. ℥j.

Pulv. gallarum . . . . ʒj.

—— opii . . . . . . ℈j. m. ft. unguentum.

In several instances we have found very good effects from the local application of port wine to the hæmorrhoidal tumours, especially where there is some degree of procidentia ani. An important measure in mitigating the pains of hæmorrhoids is pressure. No pile, in fact, ought to be allowed to protrude, and thus become strangulated. On the very first appearance of an hæmorrhoidal tumour it should be pressed back within the sphincter, and prevented from protruding by a very tight bandage and pad. By this application we have enabled men to walk about with ease, in a few minutes, who were before unable to get off the sofa. When the hæmorrhoidal attack is over, and not before, bougies may be employed.

The section on proctica exania, or procidentia ani, is replete with information. Dr. Good, however, has not apparently availed himself of an able paper on relaxed rectum by Mr. Chevalier, in one of the late volumes of the Medico-Chirurgical Transactions.*

The second order of the present class is entitled "Slanchnica," or diseases affecting the collatitious viscera. It comprises four genera,—Icterus,—Melæna,—Chololithus, (gall-stones)—Parabysma (visceral turgescence.)

The inquiry respecting the derivation of various names given to icterus by the ancients, is not worth our notice; and the question as to the real or exact use of the bile, will not, we fear, be settled for a few years to come. We do not see, however, how this question can be very intimately connected with the pathology and treatment of icterus.

The immediate cause of jaundice, all must allow to be, an obstruction to the flow of bile, from the liver to the intestines—its retrograde passage into the blood, whether by absorption or regurgitation, being merely an epiphenomenon which has given name to the disease. The most simple cause of obstruction, though we apprehend it is a very frequent one, is viscidity of the bile itself clogging the ducts through which it passes. This form generally comes on more slowly and insidiously than other forms, with the usual symptoms of langour, dyspepsy, yellow urine, torpid bowels, and clay-coloured stools. The eyes and then the skin assume the yellow tinge, there being little or no pain in the right hypochondrium, and rather a nausea than actual sickness of stomach. Vomiting, by antimonial emetics, in an early stage of the disease, is often of essential service in emulging the biliary ducts, and clearing away obstructions to the free discharge of the bile. Our author relates some cases, in detail, illustrative of this species of icterus, which are interesting enough in themselves, but we think they are rather misplaced in an elementary work, which should very rarely occupy space with the circumlocutory details of individual

---

* See p. 465 of the 1st volume of the *Analytical* Series of this Review.

cases, unless of a very rare or extraordinary nature. We may just remark here that Dr. Good occasionally commits what we might call pleonisms of expression, and which we did not expect in so learned and classical a writer. For instance, at page 367, we have this sentence—"The attention of the patient's friends was directed exclusively to the *organ of the lungs.*" We ask what is the *organ* of the lungs but the lungs themselves? Should it not have been the "organ of respiration?" Would it not be improper to say, "the organ of the liver," or the "organ of the stomach?"

The 2d species, Icterus Cololithicus, or jaundice from gall-stones, is so closely connected with the genus cololithus itself, as to have rendered it hardly worth Dr. Good's while to separate them. Indeed he only gives the definition of the 2d species, and then refers to the genus gall-stone.

We next come to Icterus Spasmodicus, where, according to our author, "the course of the bile is obstructed by a spasmodic constriction in the course of the bile-ducts." Before making any remark of our own we shall give Dr. Good's description of the symptoms.

> "The disease is ushered in by a sense of fullness at the stomach, accompanied with great languor and nausea; a violent pain at the pit of the stomach soon succeeds, with an almost incessant sickness, and an utter inability of retaining either food or medicine of any kind. The pain grows intolerable, and shoots towards the left shoulder, or spreads round the loins, and girds them as with a cord. The epigastric region is greatly distended, and cannot endure the pressure of the hand; while the pulse exhibits little variation.
>
> "The bowels are for the most part costive and moved with difficulty; the urine soon evinces a deep saffron tint, and the sooner in proportion to the violence of the other symptoms, and especially of the retching; and the surface of the body, and especially the fine sclerotic coat of the eye, assumes the same livery. And if the disease become chronic, the yellow dye is not confined to the skin or even to the fluids, but pervades every part of the body, the most compact as well as the most porous; so that the pericardium, the heart, the peritoneum, the meninges, the substance of the brain, the cartilages, and even the bones, are clothed with the common colour. Stoll, Lieutaud, Bartholin, and Morgagni, give various examples of this; though the last observes, that a yellow tinge of the brain is a rare occurrence." 374.

Now Dr. Good acknowledges that these are the symptoms of *cololithus meens*, or passing gall-stones; but, says he, "the causes and mode of treatment are different." Yet Dr. Good does not offer us any proof that the causes are different —and we venture to affirm that every practical physician, on coming to a patient with the foregoing symptoms, would pronounce the jaundice to be from *biliary concretions*, of whatever composition, obstructing the egress of bile from the liver, and thus causing all the phenomena above described. We believe that the ducts of the liver are occasionally constricted from spasm, but that the abovementioned symptoms never occur from such cause alone. If spasm continues long enough to give time for biliary concretions to form, then we have the painful phenomena above described taking place during their expulsion.

Dr. Good believes that jaundiced patients occasionally see objects yellow. This was the case in his own person; but he supposes it only happens when the humours as well as the sclerotic coat of the eye are tinged. The case is certainly a very rare one, notwithstanding the assertion of Lucretius that it is common.

> " Multaque sunt oculis in eorum denique mixta,

> " Quæ contage suâ palloribus omnia pinxit."*

The following is our author's methodus medendi.

" In the treatment of this species, emetics and cathartics, so highly beneficial in icterus *cholœus*, are of doubtful advantage. Where we have strong reason to suspect that acrimonious materials have formed a lodgment in the ducts or alvine canal, they will prove useful by evacuating them: but in all other cases they must add to the disease by increasing the irritation, and should give way to blood-letting, if the patient be in vigorous health, succeeded by opiates, the warm bath, or warm and anodyne fomentations applied to the epigastrium. The opiate should be given in pills, for the stomach will often for many hours reject liquids of every kind. Two or three grains of the extract of opium may be tried at first, and if this be insufficient, the same or even a larger dose should be repeated half an hour afterwards, and continued till the pain abates. Blistering the seat of pain has been advised by many; and I have often tried it, but without any decided effect. If useful at all, it is rather in preventing a return of the paroxysm than in shortening or mitigating it when present; and will hence be most advantageously resorted to in the interval." 377.

Over biliary concretions we imagine we have very little power. Their expulsion is a work of Nature: but there are many remedies which prevent the formation of these obstructions, by keeping up a due and a healthy secretion of the liver. Of these, the principal are gentle mercurial aperients—the taraxacum—and the nitro-muriatic acid bath—" a remedy which," says Dr. Good, " has, of late years, excited great attention, and is now surmounting an ungenerous prejudice that was at first very extensively raised against it." We have

---

* De Rerum Natura, IV. 333.

had several proofs in our own practice, of its powers as a preventive of biliary obstructions, and Dr. Good adds his testimony to the same effect. "In two or three instances the advantage has been decisive; and the patients, who had hitherto been seldom two months without a severe return of the complaint, have entirely escaped, and apparently lost, the morbid predisposition." In a few cases it entirely failed in our author's hands.

Of the 4th species of jaundice—that resulting from a scirrhous, tuberculous, or other diseased state of the liver itself, we need say little. The jaundice is a mere symptom or effect of the disease, which too often sets medicine at naught. "Mussabat tacito medicina timore." We shall have another opportunity of alluding to this subject when we come to hepatitis.

The Genus (II.) Melena, is characterized thus:—"The colour of the eyes and skin yellow-green, fuliginous, leaden, or livid; the dejections pale, occasionally dark-coloured; anxiety; depression of spirits." This is one of those diseases which have puzzled nosologists as to its place in their systems. Dr. Cullen has omitted it altogether in his Synopsis—briefly noticed it in his First Lines, under hæmatemesis, and, also, diarrhœa—and afterwards introduced it into his "Catalogue of Diseases Omitted, but which ought not to have been Omitted." It is divided by Dr. Good into two species—the cholea, and cruenta. The first, which is the black and green jaundice of authors, is thus defined. "Occasional dejections of dark or pitchy bile, intermixed with the fæces; occasional vomitings of yellowish-green and acid colluvies; great languor; often vertigo; hypochondria free from pain, but tender upon pressure." We greatly fear, that Dr. Good has not entirely settled the question of its pathology as follows:

"The liver is here evidently diseased in its structure, and a morbid deep-coloured bile, fulvous, greenish, or fuliginous, is secerned, instead of the natural excretion; from general hebetude and a want of the ordinary propulsive power, it lingers in the biliary passages, if it get into them at all; the finer part of the fluid is first absorbed, and afterwards the grosser; and what remains becomes still more viscid, more stagnant, and of a deeper hue." 389.

The green jaundice occurs more frequently in men than in women—is generally accompanied with disease of the liver, and tenderness on pressure there—pulse natural, or slower than in health—stools irregular, being sometimes pale, sometimes pitchy—urine deeply loaded—appetite irregular. The disease is generally fatal—though its progress is sometimes slow, the patient dragging out a painful existence for three, four, or five years. The treatment is undecided. Dr. Baillie, who has written a valuable paper on this subject, thinks he has found neutral salts taken daily as an aperient, of palliative use; but of a radical cure he seems to despair. Dr. Good has found mercury, combined with antimonials, "highly beneficial," particularly the plummer's pill. To these may be added, alkalies and bitter tonics, and the nitro-muriatic acid, or chlorine bath.

The second species, Melena Cruenta, or "black-vomit," is thus characterized by Dr. Good. "Occasional vomitings and dejections of grumous blood, intermixed with dark-coloured bile; pungent, tensive pain in both hypochondria; compressive pain at the pit of the stomach, and fainting."

"In this species the organs subservient to the formation of bile are in a more debilitated and decayed condition than in the preceding; and it may hence be contemplated as a disease compounded of melæna *cholæa* and hæmatemesis *passiva*, or passive hemorrhage from the vessels of the liver, spleen, or both." 392.

We think it is far from certain what is the source of the melenic discharge. Dr. Good has quoted Drs. Home and Marcard, but he has not noticed a valuable paper on melena by Dr. Cheyne, published in the first volume of the Dublin Hospital Reports, and of which we gave a full account in the first volume of our Quarterly Series, for October 1818, page 200 *et seq*. Dr. Cheyne, in that paper, draws the attention of the profession to the "alternate excess of morbid action in the mucous and serous membranes," by highly illustrative examples of the transference of disease from one to the other of these structures. The disease, whether symptomatic (as in the black vomit of fever) or idiopathic, is extremely dangerous, and no fixed principles of treatment are known.

Genus III. is *Chololithus*, or gall-stone, the symptoms of which are those of icterus spasmodicus. In the disease before us we find certain portions of the bile indurated, and assuming a concrete, often a crystallized form, sometimes of a laminated structure, evincing a tendency towards crystallized rays in the centre, with concentric laminæ towards the surface. Fourcroy supposed these concretions were a resinous matter, combined with a peculiar oil, and a certain quantity of albumen, forming three of the constituent principles of bile. All these principles have been denied by Berzelius, who has discovered that bile becomes resinous only in the process of experiment, by supersaturating it with acids, while the material hitherto regarded as albumen, is nothing more than a small portion of mucus furnished from the gall-bladder! We fear that many supposed constituents of animal and other matters are the "products of experiment," the same as in the above instance. We know, however, that gall-stones are inflammable—that they are found of all sizes in the gall-bladder and ducts—that they are passed with great pain—that they will sometimes lie quiet in the cyst for years, without inconvenience—and that they will occasionally endanger or even destroy life, if lodged long in the ductus communis. Of the treatment we have already spoken.

"Our best and wisest exertions, therefore, must be of a palliative kind, with a view of easing and quickening the passage of the gall-stone. We have no direct means, however, of doing the last: and all we can hope to accomplish is that of rendering a little collateral assistance to the expulsive efforts which are made by nature herself. The duct becomes dilated by the circumambient pressure of the concretion as it gradually passes forward, urged on by the same action that propels the bile in a state of health. Vomiting, therefore, by compressing the whole abdominal viscera, and, particularly, the full and distended gall-bladder and biliary vessels, may afford one mean of pushing forward the concretion: but a gentle force, and consequently gentle vomits, will promise fairer than those which act violently. Dr. Darwin affirms, that in two instances he saw from thirty to fifty gall-stones voided after taking only an oil vomit. If the patient be of tolerable vigour, and inflammation be apprehended, bleeding should precede the exhibition of emetics. Cathartics, by exciting the action of the intestines, and directly stimulating the mouth of the common bile-ducts, contribute, also, to excite action through its entire range, and thus farther favour the expulsion of the concretion. And as we often find its passage evidently opposed by spasmodic constriction, opium, given very freely and repeated every hour or two, and relaxing the skin by fomentations or the warm bath, will in such cases be of essential service. Horse exercise cannot always be made use of: but where it can be submitted to, it is one of the best auxiliaries we can recommend." 402.

The last genus in the present order is Parabysma, hitherto denominated physconia, but now exchanged for a genuine Greek term, (from παραβυω,) signifying morbid congestion, coacervation, or infarction—"a monstrous race of diseases," as our author expresses it, "which we can rarely hope to conquer, unless we have an opportunity of strangling them in their infancy." This genus comprehends enlargements of the liver, spleen, pancreas, mesentery, intestines, and omentum, together with a complication of some or several of these. We shall notice but one or two of them. The parabysma hepaticum arises from various causes, and is attended with a diversity of symptoms, which but seldom enables us to ascertain the exact nature of the swelling ante obitum.

Speaking of the species parabysma hepaticum, helminthicum, or hydaticum, Dr. Good observes—

"As this species of the parabysma depends almost entirely on an atony of the liver, the intumescence increases in many instances in proportion to that atony, and particularly when debility of the liver is combined with a general debility of the entire system. And hence the liver is frequently known to enlarge in proportion as every other organ becomes torpid and decays. On which account the liver is often found of an enormous size in dropsical patients. Mr. Gooch gives a case, in which, during dropsy, it acquired the monstrous weight of six hundred and twenty-eight pounds.* Baldinger reports another instance in which it reached six hundred and twenty pounds;† and Bonet, a third, in which it weighed only two pounds less.‡" 408.

These amazing enlargements of the liver have escaped our notice, and we have not Gooch's or Baldinger's cases to refer to. On turning to Bonetus, we observe that lib. 1. sect. xviii. is "de oculorum affectibus," and consequently there was no hope of finding enlargements of the liver there. The 16th section of the third book, however, "de tumore hypochondriorum," presents us with many remarkable instances of hepatic intumescence, of which the following, from the language employed, would appear to be the most extraordinary which ever occurred to Bonetus. "Cum itaque opus aggrederer, et circa parenchyma hepatis, tumoris indagandi gratia occuparer, illud cum stupore et admiratione tam vastum ac mole ponderosum videns adstantibus monstro, qui perterriti vix oculis fidem habebant. De pondere autem certior fieri volens, monebam chirurgum ut extraheret, et totum scirrhosum repertum, pendebat libras xiv. civiles."—*Lib.* III. *Sect.* xvi. *Obs.* 3.

We do not, for a moment, question the accuracy of Dr. Good; but when we consider that a liver, of the size noticed in the extract, must have weighed nearly three stone more than the *whole* of Lambert, "the *greatest* man in England," as the bills facetiously expressed it, we cannot but doubt exaggeration on the part of the original narrators.

We shall not notice the parabysma splenicum, as a professed work on diseases of the spleen is in the hands of one of our coadjutors for early review. And here we must close our first article on the important work before us. The principal defects which we have noticed in this extended publication are the two following:—1st. Dr. Good has not, on

* Med and Surg. Obs. † N. Magaz. Band. vii p. 275.

‡ Sepulcr. Lib. I. Sect. xviii.

several occasions, brought down his pathological information to the most recent periods—a duty incumbent on every one who undertakes a work of this description. 2dly. He occasionally introduces obsolete, irrelevant, or useless materials, when the paramount object ought to be to fill every point of space with the very best, and nothing but the best portions of knowledge. With these trifling defects, which another edition will easily rectify, we have no hesitation in pronouncing the work, beyond all comparison, the best of the kind in the English language. With the naval, the military, the provincial, and the colonial practitioner, the work before us ought, at once, to supersede the unscientific compilation of Dr. Thomas—and it will do so.

---

